# A Missense Variant in *COMT* Associated with Hearing Loss among Young Adults: The National Longitudinal Study of Adolescent to Adult Health (Add Health)

**DOI:** 10.3390/biomedicines10112756

**Published:** 2022-10-31

**Authors:** Chuan-Ming Li, Le Chen, Guanjie Chen, Jianhua Zhang, Howard J. Hoffman

**Affiliations:** 1 Epidemiology and Statistics Program, Division of Scientific Programs, National Institute on Deafness and Other Communication Disorders (NIDCD), National Institutes of Health (NIH), Bethesda, MD 20892, USA; 2 Center for Research on Genomics and Global Health, National Human Genome Research Institute, National Institutes of Health (NIH), Bethesda, MD 20892, USA; 3 Metabolic Disease Branch, National Institute of Diabetes and Digestive and Kidney Diseases (NIDDK), National Institutes of Health (NIH), Bethesda, MD 20892, USA

**Keywords:** add health, catechol-O-methyltransferase (COMT), hearing loss

## Abstract

Hearing loss is a major public problem with a heritability of up to 70%. Catechol-O-methyltransferase (COMT) encodes an enzyme that is highly expressed in sensory hair cells of the inner ear. The association between *COMT* and hearing loss has not been reported previously in nationally representative population-based studies. A regression linear model was used to estimate associations between the allele/genotype of *COMT* and self-reported hearing loss based on 13,403 individuals from Wave IV of the Add Health study, a nationally representative sample of multiethnic U.S. young adults. The inverse variance-weighted effect magnitude was estimated using a genetic meta-analysis model. The “A” allele frequency of rs6480 (a missense variant in *COMT*) was 0.44. The prevalence of hearing loss was 7.9% for individuals with the “A” allele and 6.5% for those with the “G” allele. The “A” allele was significantly associated with increased hearing loss (*p* = 0.01). The prevalence of hearing loss was 6.0%, 7.2%, and 8.7% for individuals with GG, AG, and AA genotypes, respectively, which was consistent with a genetic additive model. The genotypic association model showed that rs4680 was significantly associated with increased hearing loss (*p* = 0.006). A missense variant of rs4680 in *COMT* was significantly associated with increased hearing loss among young adults in a multi-racial/ethnic U.S. population-based cohort.

## 1. Introduction

Hearing loss is a major public health problem with an estimated 1.57 billion people worldwide with hearing loss in 2019, accounting for one in five people. It was the third largest cause of global years lived with disabilities (YLD) and the leading cause of global YLDs for adults older than 70 years [1]. Hearing loss does not only impact communication, it is also associated with social isolation, loneliness, depression, cognitive decline, dementia, falls, and other health conditions [2,3,4,5,6]. The total global economic costs of hearing loss exceed $981 billion [7].

The etiology of hearing loss is multifactorial and includes genetic factors, environmental factors, and their interaction. Many genetic variants are associated with hearing loss [8,9]. The heritability of adult-onset hearing loss has been estimated to be 36–70% [9,10,11,12,13,14].

We examine the association of hearing loss with selected allelic haplotypes. For example, catechol-O-methyltransferase (COMT) encodes an enzyme that is involved in the inactivation of catecholamine neurotransmitters and is highly expressed in sensory hair cells of the inner ear [15]. COMT has been reported as being essential for auditory function [15]. The rs4680 (*COMT*) allele is nonsynonymous with a guanine (G) to adenine (A) substitution in the DNA nucleotide sequence resulting in a valine (Val) to methionine (Met) amino acid substitution. rs4680 G > A influences COMT enzyme activity (AA with low, AG with medium, GG with higher enzyme activity), which catalyzes the transfer of a methyl group from S-adenosylmethionine to catecholamines that play a key role in auditory function [15]. A genetic, intronic variant (rs9332377) in *COMT* has been associated with cisplatin-induced hearing loss in children [16,17]. Both SNPs rs4680 and rs9332377 (~4 kb) are in linkage disequilibrium with a D-prime score of 0.84 in the 1000 genomes project (www.internationalgenome.org). The association between rs4680 and hearing loss has not been reported previously in population-based studies.

The self-reported hearing loss questions in the Add Health Study provide a useful measure for genetic studies [9,11,18]. In Wave IV of the Add Heath study, three single nucleotide polymorphisms (SNPs) of rs12945042 (serotonin transporter), rs1800497 (dopamine D2 receptor, DRD2), and rs4680 (COMT) were ascertained along with self-reported hearing loss information. Prior studies have shown that the serotonin transporter (SERT) is an important marker of the status of serotonergic neurons, is expressed in the central auditory pathway, and plays a role in the auditory process [19]. Dopamine is present in the first synaptic complex of the auditory pathway. Additionally, it has been shown that sulpiride, an antagonist of the D2 dopamine receptor, can lead to an attenuation of tinnitus perception [20]. In this report, we evaluate associations between genetic factors and hearing loss in the multi-racial/ethnic U.S. population.

## 2. Materials and Methods

### 2.1. Participants and Hearing Loss

This study was based on data collected from Add Health, an ongoing, nationally representative longitudinal study, which covered the social, behavioral, and biological linkages in health and developmental trajectories from early adolescence into adulthood [21]. The adolescents of the Add Health cohort have been followed for more than 20 years since Wave I in 1995 when the adolescents were in grades 7–12, followed by Wave II in 1996, Wave III in 2001-02 when the participants were aged 18–26, Wave IV in 2008 when they were aged 24–32, and most recently Wave V in 2016–2018 when they were aged 32–42. The data from Wave IV are used in this report. All participants gave informed consent, and the study was approved by the Institutional Review Board of the University of North Carolina at Chapel Hill.

The question was, “Which statement best describes your hearing without a hearing aid or other assistive devices? Response options: 1. Excellent; 2. Good; 3. A little trouble; 4. Moderate hearing trouble; 5. A lot of trouble; 6. Deaf.” Hearing loss was defined by any of the following four responses: “a little trouble”, “moderate hearing trouble”, “a lot of trouble”, and “deaf”.

### 2.2. DNA Sample

Biological specimens (saliva) in Wave IV of the Add Health study were collected from a large, nationally representative sample of young adults by trained and certified field interviewers. Salivary buccal cell lysis and DNA stabilization were performed in the field and shipped to a central lab for DNA extraction, genotyping, and archiving [22]. The collection of capillary whole blood was also processed [23]. Three single nucleotide polymorphisms (SNPs), rs1800497 (Dopamine D2 receptor TaqIA (DRD2), 11:113270828), rs4680 (a missense variant in COMT, 22:19951271), and rs12945042 (near SLC6A4, Serotonin Transporter, 17:28571928) [24], were genotyped. The rationale, equipment, protocol, genotyping, data cleaning, quality, and other measures were based on additional genotyping of salivary buccal cell DNA. A description of the assay of dried capillary whole blood spots can be found at https://addhealth.cpc.unc.edu/documentation/user-guides/ (accessed on 19 October 2022).

### 2.3. Genotype

All SNP assays were conducted on either an Applied Biosystems TaqMan^®^ OpenArray^®^ or Illumina BeadXperss^®^ GoldenGate^®^ platform. The Hardy Weinberg equilibrium (HWE) was evaluated for each allele genotype in race/ethnicity-specific strata. HWE *p* value < 0.05 and Minor Allele frequency (MAF) < 0.01 were used for QC.

### 2.4. Association Testing

To evaluate the association between hearing loss and the SNP allele/genotype, a logistic regression model was performed, adjusting for sex, age, family income, smoking status, and education levels. Three genetic models (dominant, recessive, and additive) were used to evaluate the genotypic association, respectively. The association testing was performed by racial/ethnic groups. Before the logistic regression, we examined the data for influential observations and the presence of variance inflation. No influential observations or multicollinearity was found. The R package of meta was used for meta-analysis with an inverse variance-weighed fixed effects method [25] in combined cohorts.

### 2.5. Protein Structure and Function Predictions

From a paradigm of protein sequence-to-structure-to-function, we uploaded translated sequences (amino acid sequences) to the I-TASSER online server (https://zhanggroup.org//I-TASSER/, accessed on 19 October 2022) [26,27,28] to predict the three-dimensional (3D) protein structure. We used the protein 3D structure to find matches in a protein function database in order to predict protein functions [29]. PyMol [30] was used to view and analyze the protein structure.

## 3. Results

### 3.1. Characteristics of Participants

A total of 13,403 (non-Hispanic White 57.8%, non-Hispanic Black 20.6%, Hispanic 16.1, and non-Hispanic Asian 5.6%) adults were included in the study (Table 1). The prevalence of reported hearing loss in the non-Hispanic White group (9%) was significantly higher than in other racial/ethnic groups (*p* < 0.001). The prevalence of hearing loss in males was significantly higher than in females. Participants with a higher education had a significantly lower prevalence of hearing loss (*p* < 0.001), and, concomitantly, those with a lower household income had a significantly higher prevalence of hearing loss (*p* < 0.001, Table 1). In addition, participants who had ever smoked regularly had a higher prevalence of hearing loss (~10%, *p* < 0.0001).

### 3.2. Allele Frequency and Association with Hearing Loss

Minor allele frequencies (MAF) were 0.45 (“A”), 0.26 (“A”), and 0.29 (“T”) for rs4680, rs1800497, and rs12945042, respectively. The prevalence of hearing loss for individuals who carried minor alleles was higher than for individuals who carried major alleles (Table 2) for rs4680 and rs12945042. For rs1800497, the prevalence of hearing loss for individuals who carried the G allele (0.74) was higher.

The rs4680 (missense in *COMT*) was significantly associated with a higher prevalence of hearing loss (β (95% CI) = 0.13 (0.04, 0.23), *p* = 0.010, Table 3). The results adjusted for racial/ethnic-specific groups showed a stronger effect—higher β estimates—for non-Hispanic Asians compared to the other three groups, albeit the number of non-Hispanic Asians in the population sampled was smaller, which constrained the influence on the combined (meta-analysis) for both the allele (A vs. G) and genotype/additive models. No associations were identified for rs1800497 and rs12945042 (Table S1).

### 3.3. Frequency of Genotype and Genotypic Association with Hearing Loss

**Figure 1:**
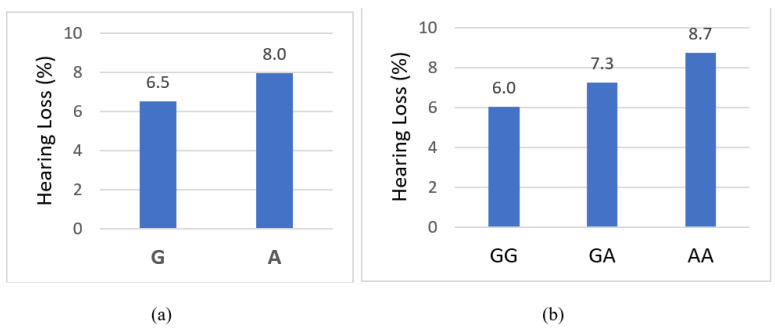
Prevalence of hearing loss by allele and genotype of rs4680 (a missense variant in catechol-O-methyltransferase): (**a**) allele; (**b**) genotype.

The prevalence of hearing loss by allele and genotype is presented in Figure 1a,b for rs4680, Figure S1 for rs1800497 and Figure S2 for rs12945042. The prevalence of hearing loss for individuals who carried “GG”, “GA”, and “AA” genotypes in rs4680 was 6.0%, 7.3%, and 8.7%, respectively, which conforms with a genetic additive model. The rs4680 (effective allele A) is significantly associated with a higher prevalence of hearing loss (*p* = 0.0061, Table 3). No genotypic associations for rs1800497 and rs12945042 were identified (Table S1). Additionally, no gene-gene or gene-environment interactions were observed.

### 3.4. COMT Protein Structure

**Figure 2:**
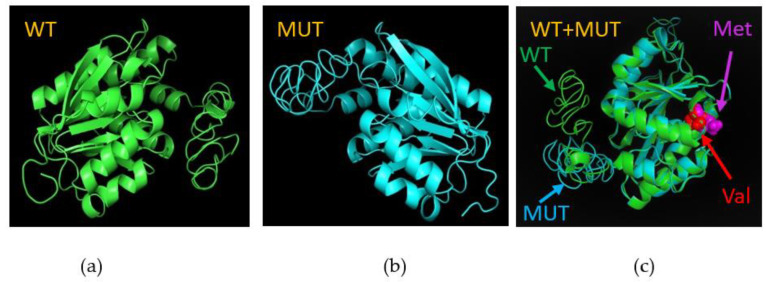
The wild type and mutation (rs4680) of COMT protein structure: (**a**) wild type; (**b**) mutation; (**c**) aligned WT and mutation. Abbreviations: WT: wild type; MUT: mutation; Met: methionine; Val: valine.

The variant rs4680 (COMT) is a missense variant from a guanine (G) to adenine (A) substitution in the DNA nucleotide sequence resulting in a valine (Val) to methionine (Met) amino acid substitution at the position 158. I-TASSER predicted the possible protein structure based on an amino acid sequence with V158M. The predicted protein structure was different from the wild type (Figure 2a–c) with a moderate change in the protein structure.

## 4. Discussion

Add Health is an ongoing, nationally representative longitudinal study of social, behavioral, and biological factors in health and developmental trajectories from early adolescence into adulthood. The participants in Wave IV were 24–32 years old. We analyzed associations between hearing loss and three measured single nucleotide polymorphisms (SNPs) in three candidate genes. We found that rs4680 (catechol-O-methyltransferase, *COMT*, Gene ID: 1312) is associated with an increased prevalence of hearing loss.

*COMT* encodes an enzyme that is involved in the inactivation of catecholamine neurotransmitters and is highly expressed in sensory hair cells of the inner ear [15]. In addition, rs4680 (*COMT*) is nonsynonymous with a guanine (G) to adenine (A) substitution in the DNA nucleotide sequence resulting in a valine (Val) to methionine (Met) amino acid substitution. rs4680 G > A influences COMT enzyme activity (AA with low, AG with medium, GG with higher enzyme activity) [31,32]. A major function of COMT is to regulate dopamine levels that influence the processing of auditory signals within the mammalian cochlea, which is therefore directly linked to the function of sensory hair cells [15]. An enzyme, LRTOMT2, has a 60% similarity with COMT (212 conserved amino acids including the substrate-binding region) and functions as a catechol-O-methyltransferase, which has been shown to be essential for auditory function in mice and humans [15]. Two intron variants (rs4646316, rs9332377) and their haplotypes are associated with cisplatin-induced hearing loss [17]. The haplotypes rs4646316 G and rs9332377 A carry a low-activity synonymous *COMT* variant, rs4818, which has an association with cisplatin-induced hearing loss and confers an 11-to-18-fold reduction in COMT protein levels due to alterations in the mRNA secondary structure [33]. The missense variant of rs4680 is in 62 base pairs with rs4818 (synonymous variant), and both are in a strong LD with r^2^~0.70 [34]. This suggests that the hearing loss may be related to increased S-adenosylmethionine through a reduced COMT activity [17].

Following the discovery of genetic association, function studies are a critical next step. Based on the sequence-to-structure-to-function paradigm, I-TASSER was developed as an online platform for protein structure and function prediction [28]. The identified genetic variant in this study was missense. Amino acid sequences of both wild and mutation types in V158M were uploaded onto the I-TASSER server to predict protein structures and were aligned in PyMOL to compare wild-mutation protein structures. The different protein structures between wild and mutation in V158M (rs4680, COMT) led to predicted changes in the protein function, which could result in an altered COMT activity. To verify this hypothesis, eQTL analysis was performed in 268 liver biopsy samples and 16 SNPs in COMT, which showed that rs4680 G > A had the strongest association, explaining 20.2% of the variance in the level of activity [35].

One potential limitation of this study is that self-reported hearing loss was used to estimate the association with a missense variant in *COMT*. Importantly, the self-reported hearing loss question used in the Add Health study is identical to the phrasing of the question on reported hearing loss that is used and analyzed in other major U.S. population-based health surveys [4]. In population-based studies, self-reported hearing loss is much more commonly available than audiometric threshold measures. The use of self-reported hearing loss as an appropriate phenotypic measure has been demonstrated [9]. Investigations based on audiometric thresholds of hearing loss may be conducted in the future to replicate the findings in this report.

In summary, we analyzed data from Wave IV of Add Health, a population-based longitudinal cohort study, and identified a missense variant, rs4680 G > A, which was significantly associated with an increased prevalence of hearing loss. Compared to the wild type, this missense variant led to a protein structure change that may cause hearing loss through an increase in S-adenosylmethionine levels resulting from a reduced COMT activity.

## Figures and Tables

**Table 1:** Prevalence of hearing loss by socio-demographic characteristics and smoking status.

		N (%)	Hearing Loss (%)	*p* Value
**All**		13,403	7.2	
**Age (years)**				0.3223
	<29	4969 (37.1)	6.9	
	≥29	8434 (62.9)	7.3	
**Sex**				<0.0001
	Male	6226 (46.5)	8.5	
	Female	7177 (53.5)	6.1	
**Race/Ethnicity**				<0.0001
	Non-Hispanic White	7742 (57.8)	9.0	
	Non-Hispanic Black	2757 (20.6)	4.8	
	Hispanic	2156 (16.1)	4.5	
	Non-Hispanic Asian	748 (5.6)	4.8	
**Education**				<0.0001
	≤high school	3278 (24.5)	8.6	
	≤college	6004 (44.8)	8.0	
	More than college	4120 (30.7)	4.8	
**Household income**				<0.0001
	<$25,000	2106 (16.7)	8.8	
	$25,000–<$50,000	3545 (28.1)	8.1	
	$50,000–<$75,000	3076 (24.4)	6.8	
	$75,000–$100,000	1901 (15.1)	5.8	
	≥$100,000	1976 (15.7)	5.3	
**Ever smoked regularly**				<0.0001
	Yes	5612 (48.9)	10.1	
	No	6986 (51.1)	5.7	

**Table 2:** Frequencies of allele and genotype in rs4680, re800497, and rs12945042 and prevalence of hearing loss.

		rs4680 (*COMT*)			rs800497 (*DRD2*)			rs12945042 (near *SLC6A4*)	
	Allele/Genotype	N (%)	Hearing loss (%)	Allele/Genotype	N (%)	Hearing loss (%)	Allele/Genotype	N (%)	Hearing loss (%)
**Allele**									
**All**	A	11,962 (44.6)	7.9	A	6963 (26.2)	6.4	C	18,589 (71.9)	7.1
	G	14,844 (55.4)	6.5	G	19,561 (73.78)	7.4	T	7255 (29.1)	7.5
**Non-Hispanic White**	A	7773 (50.2)	9.5	A	3361 (21.9)	8.7	C	10,322 (69.1)	9.2
	G	7711 (49.8)	8.5	G	11,961 (78.1)	9.0	T	4622 (30.9)	8.8
**Non-Hispanic Black**	A	1864 (33.8)	5.0	A	1792 (32.9)	4.6	C	4133 (78.1)	4.7
	G	3650 (66.2)	4.7	G	3662 (67.1)	4.9	T	1157 (21.9)	5.4
**Hispanic**	A	1867 (43.3)	4.8	A	1311 (30.7)	4.0	C	2999 (72.1)	4.3
	G	2445 (56.7)	4.3	G	2959 (69.3)	4.7	T	1159 (27.9)	5.2
**Non-Hispanic Asian**	A	458 (30.6)	6.8	A	499 (33.8)	4.4	C	1135 (78.1)	4.2
	G	1038 (69.4)	4.8	G	979 (66.2)	5.1	T	317 (21.8)	6.3
**Genotype**									
**All**	AA	2761 (25.1)	8.7	AA	1013 (7.3)	5.9	CC	6788 (51.8)	6.9
	AG	6440 (48.6)	7.2	AG	5260 (37.8)	6.5	CT	5264 (40.1)	7.6
	GG	4202 (26.3)	6.0	GG	7634 (54.9)	7.6	TT	1050 (8.0)	7.3
**Non-Hispanic White**	AA	1944 (25.1)	9.9	AA	387 (4.8)	8.5	CC	3622 (47.8)	9.2
	AG	3885 (50.2)	9.0	AG	2716 (34.1)	8.6	CT	3224 (42.5)	9.0
	GG	1913 (24.7)	8.0	GG	4869 (61.1)	9.2	TT	733 (9.7)	8.0
**Non-Hispanic Black**	AA	321 (11.6)	5.6	AA	300 (10.3)	4.3	CC	1622 (60.5)	4.6
	AG	1222 (44.3)	4.7	AG	1296 (44.6)	4.5	CT	942 (35.2)	5.2
	GG	1214 (44.0)	4.6	GG	1309 (45.1)	4.9	TT	115 (4.30	5.4
**Hispanic**	AA	421 (19.5)	6.2	AA	233 (10.4)	4.3	CC	1090 (51.7)	3.8
	AG	1025 (47.5)	3.6	AG	903 (40.2)	3.5	CT	857 (40.6)	5.5
	GG	710 (32.9	4.8	GG	1112 (49.5)	4.9	TT	163 (7.7)	4.3
**Non-Hispanic Asian**	AA	75 (10.0)	6.7	AA	93 (11.9)	4.3	CC	454 (61.8)	4.2
	AG	308 (41.2)	6.8	AG	345 (44.1)	5.2	CT	241 (5.3)	5.4
	GG	365 (48.8)	2.7	GG	344 (44.0)	5.2	TT	734 (5.3)	10.3

**Table 3:** Associations between hearing loss and allele and genotype of rs4680.

SNP	Model *		β Estimates (95% CI)	Weight
**rs4680**	**Allele (A vs. G)**			
	**—**	Non-Hispanic White	0.11 (−0.01–0.22)	72.9%
		Non-Hispanic Black	0.11 (−0.16–0.39)	12.9%
		Hispanic	0.11 (−0.20–0.42)	10.2%
		Non-Hispanic Asian	0.61 (0.11–1.10)	4.0%
		Meta-analysis	0.13 (0.04–0.23)	
		*p* value	0.0101	
	**Genotype (additive model)** (reference: GG)			
		Non-Hispanic White	0.12 (0.01–0.24)	72.9%
		Non-Hispanic Black	0.10 (−0.17–0.38)	12.8%
		Hispanic	0.11 (−0.20–0.41)	10.4%
		Non-Hispanic Asian	0.62 (0.12–1.12)	3.9%
		Meta-analysis	0.14 (0.04–0.24)	
		*p* value	0.0061	

* The allelic and genotypic association models were evaluated using regression (additive model) adjusted for age, sex, education level, smoking status, and household income by racial/ethnic groups.

## Data Availability

Public-use data consists of one-half of the core sample, and one-half of the oversample of African-American adolescents with a parent who has a college degree, chosen at random. Public-use data is available for Waves I-V. Restricted-use data will be distributed only to certified researchers (this includes researchers that are located outside of the U.S.) who demonstrate their capability of maintaining limited access and confidentiality of the restricted-use data. More information is available at https://data.cpc.unc.edu/projects/2/view.

## Supplementary Materials

The following supporting information can be downloaded at: https://www.mdpi.com/article/10.3390/biomedicines10112756/s1, Table S1: Associations between hearing loss and allele and genotype of rs1800497 and rs1800497; Figure S1: Prevalence of hearing loss by allele and genotype of rs1800497; Figure S2: Prevalence of hearing loss by allele and genotype of rs12945042.

## References

[1] GBD 2019 Hearing Loss Collaborators (2021). Hearing loss prevalence and years lived with disability, 1990–2019: Findings from the Global Burden of Disease Study 2019. Lancet.

[2] Wilson B.S., Tucci D.L., Merson M.H., O’Donoghue G.M. (2017). Global hearing health care: New findings and perspectives. Lancet.

[3] Livingston G., Sommerlad A., Orgeta V., Costafreda S.G., Huntley J., Ames D., Ballard C., Banerjee S., Alistair Burns J.C., Cohen-Mansfield J. (2017). Dementia prevention, intervention, and care. Lancet.

[4] Li C., Zhang X., Hoffman H.J., Cotch M.F., Themann C.L., Wilson M.R. (2014). Hearing impairment associated with depression in US adults, National Health and Nutrition Examination Survey 2005–2010. JAMA Otolaryngol. Head Neck Surg..

[5] Lin F.R., Yaffe K., Xia J., Xue Q., Harris T.B., Purchase-Helzner E., Satterfield S., Ayonayon H.N., Ferrucci L., Simonsick E.M. (2013). Hearing loss and cognitive decline in older adults. JAMA Intern. Med..

[6] Nachtegaal J., Festen J.M., Kramer S.E. (2011). Hearing Ability and its Relationship with Psychosocial Health, Work-Related Variables, and Health Care Use: The National Longitudinal Study on Hearing. Audiol. Res..

[7] McDaid D., Park A.L., Chadha S. (2021). Estimating the global costs of hearing loss. Int. J. Audiol..

[8] Van Camp G.S.R.J.H. Hereditary Hearing Loss Homepage. https://hereditaryhearingloss.org.

[9] Trpchevska N., Freidin M.B., Broer L., Oosterloo B.C., Yao S., Zhou Y., Vona B., Bishop C., Bizaki-Vallaskangas A., Canlon B. (2022). Genome-wide association meta-analysis identifies 48 risk variants and highlights the role of the stria vascularis in hearing loss. Am. J. Hum. Genet..

[10] Kvestad E., Czajkowski N., Krog N.H., Engdahl B., Tambs K. (2012). Heritability of Hearing Loss. Epidemiology.

[11] Cherny S.S., Livshits G., Wells H.R.R., Freidin M.B., Malkin I., Dawson S.J., Williams F.M.K. (2020). Self-reported hearing loss questions provide a good measure for genetic studies: A polygenic risk score analysis from UK Biobank. Eur. J. Hum. Genet..

[12] Wolber L.E., Steves C.J., Spector T.D., Williams F.M.K. (2012). Hearing ability with age in northern European women: A new web-based approach to genetic studies. PLoS ONE.

[13] Gates G.A., Couropmitree N.N., Myers R.H. (1999). Genetic associations in age-related hearing thresholds. Arch. Otolaryngol. Head. Neck Surg..

[14] Christensen K., Frederiksen H., Hoffman H.J. (2001). Genetic and environmental influences on self-reported reduced hearing in the old and oldest old. J. Am. Geriatr. Soc..

[15] Du X., Schwander M., Moresco E.M.Y., Viviani P., Haller C., Hildebrand M.S., Pak K., Tarantino L., Roberts A., Richardson H. (2008). A catechol-O-methyltransferase that is essential for auditory function in mice and humans. Proc. Natl. Acad. Sci. USA.

[16] Thiesen S., Yin P., Jorgensen A.L., Zhang J.E., Manzo V., McEvoy L., Barton C., Picton S., Bailey S., Brock P. (2017). TPMT, COMT and ACYP2 genetic variants in paediatric cancer patients with cisplatin-induced ototoxicity. Pharm. Genom..

[17] Ross C.J.D., Katzov-Eckert H., Dubé M., Brooks B., Rassekh S.R., Barhdadi A., Feroz-Zada Y., Visscher H., Brown A.M.K., Rieder M.J. (2009). Genetic variants in TPMT and COMT are associated with hearing loss in children receiving cisplatin chemotherapy. Nat. Genet..

[18] Wells H.R.R., Freidin M.B., Zainul Abidin F.N., Payton A., Dawes P., Munro K.J., Morton C.C., Moore D.R., Dawson S.J., Williams F.M.K. (2019). GWAS Identifies 44 Independent Associated Genomic Loci for Self-Reported Adult Hearing Difficulty in UK Biobank. Am. J. Hum. Genet..

[19] Kang H., Wang C., Chen H., Li I., Cheng C., Liu R., Huang W., Shiue C., Ma K. (2013). Investigating the effects of noise-induced hearing loss on serotonin transporters in rat brain using 4-[18F]-ADAM/small animal PET. Neuroimage.

[20] Wang K., Tang D., Ma J., Sun S. (2020). Auditory Neural Plasticity in Tinnitus Mechanisms and Management. Neural Plast.

[21] Harris K.M., Halpern C.T., Whitsel E.A., Hussey J.M., Killeya-Jones L.A., Tabor J., Dean S.C. (2019). Cohort Profile: The National Longitudinal Study of Adolescent to Adult Health (Add Health). Int. J. Epidemiol..

[22] Entzel P., Whitsel E., Richardson A., Tabor J., Hallquist S.P., Hussey J., Halpern C., Harris K. Add Health Wave IV Documentation: Report Cardiovascular and Anthropometric Measures 2009. https://addhealth.cpc.unc.edu/wp-content/uploads/docs/user_guides/Wave_IV_Cardiovascular_and_anthropometric_documentation.pdf.

[23] Whitsel E., Tabor J., Nguyen Q., Cuthbertson C., Wener M., Potter A.J., Killeya-Jones L.A., Harris K. (2012). Add Health Wave IV Documentation: Report Measures of Glucose Homeostasis. https://addhealth.cpc.unc.edu/wp-content/uploads/docs/user_guides/Glucose_HbA1c.pdf.

[24] Munn-Chernoff M.A., McQueen M.B., Stetler G.L., Haberstick B.C., Rhee S.H., Sobik L.E., Corley R.P., Smolen A., Hewitt J.K., Stallings M.C. (2012). Examining associations between disordered eating and serotonin transporter gene polymorphisms. Int. J. Eat. Disord..

[25] Balduzzi S., Rücker G., Schwarzer G. (2019). How to perform a meta-analysis with R: A practical tutorial. Evid. Based Ment. Health.

[26] Roy A., Kucukural A., Zhang Y. (2010). I-TASSER: A unified platform for automated protein structure and function prediction. Nat. Protoc..

[27] Yang J., Zhang Y. (2015). Protein Structure and Function Prediction Using I-TASSER. Curr. Protoc. Bioinform..

[28] Yang J., Yan R., Roy A., Xu D., Poisson J., Zhang Y. (2015). The I-TASSER Suite: Protein structure and function prediction. Nat. Methods.

[29] Chen G., Shriner D., Zhang J., Zhou J., Adikaram P., Doumatey A.P., Bentley A.R., Adeyemo A., Rotimi C.N. (2022). Additive genetic effect of GCKR, G6PC2, and SLC30A8 variants on fasting glucose levels and risk of type 2 diabetes. PLoS ONE.

[30] Alexander N., Woetzel N., Meiler J. (2011). Bcl::Cluster: A method for clustering biological molecules coupled with visualization in the Pymol Molecular Graphics System. IEEE Int. Conf. Comput. Adv. Bio Med. Sci..

[31] Lachman H.M., Papolos D.F., Saito T., Yu Y.M., Szumlanski C.L., Weinshilboum R.M. (1996). Human catechol-O-methyltransferase pharmacogenetics: Description of a functional polymorphism and its potential application to neuropsychiatric disorders. Pharmacogenetics.

[32] Schacht J.P. (2016). COMT val158met moderation of dopaminergic drug effects on cognitive function: A critical review. Pharm. J..

[33] Nackley A.G., Shabalina S.A., Tchivileva I.E., Satterfield K., Korchynskyi O., Makarov S.S., Maixner W., Diatchenko L. (2006). Human catechol-O-methyltransferase haplotypes modulate protein expression by altering mRNA secondary structure. Science.

[34] Ward L.D., Kellis M. (2012). HaploReg: A resource for exploring chromatin states, conservation, and regulatory motif alterations within sets of genetically linked variants. Nucleic. Acids Res..

[35] Zhang J., Ji Y., Moon I., Pelleymounter L.L., Salavaggione O.E., Wu Y., Jenkins G.D., Batzler A.J., Schaid D.J., Weinshilboum R.M. (2009). Catechol O-methyltransferase pharmacogenomics: Human liver genotype-phenotype correlation and proximal promoter studies. Pharm. Genom..

