# Supplementary material for: A Missense Variant in COMT Associated with Hearing Loss among Young Adults: The National Longitudinal Study of Adolescent to Adult Health (Add Health)
Source: Biomedicines. 2022 Oct 31;10(11):2756. doi: 10.3390/biomedicines10112756 (PMC9687786; doi:10.3390/biomedicines10112756)
Supplement: Supplementary file 1 [file biomedicines-10-02756-s001.zip › biomedicines-1960639-supplementary.pdf]

biomedicines

MDPI

Article

A Missense Variant in *COMT* Associated with Hearing Loss among Young Adults: The National Longitudinal Study of Adolescent to Adult Health (Add Health)

Chuan-Ming Li [1,*], Le Chen [1], Guanjie Chen [2], Jianhua Zhang [3] and Howard J. Hoffman [1,*]

1 Epidemiology and Statistics Program, Division of Scientific Programs, National Institute on Deafness and Other Communication Disorders (NIDCD), National Institutes of Health (NIH), Bethesda, MD 20892, USA
2 Center for Research on Genomics and Global Health, National Human Genome Research Institute, National Institutes of Health (NIH), Bethesda, MD 20892, USA
3 Metabolic Disease Branch, National Institute of Diabetes and Digestive and Kidney Diseases (NIDDK), National Institutes of Health (NIH), Bethesda, MD 20892, USA
* Correspondence: chuan-ming.li@nih.gov (C.-M.L.); howard.hoffman@nih.gov (H.J.H.); Tel.: +1-301-435-1351 (C.-M.L.); +1-301-435-1349 (H.J.H.)

Citation: Li, C.-M.; Chen, L.; Chen, G.; Zhang, J.; Hoffman, H.J. A Missense Variant in *COMT* Associated with Hearing Loss among Young Adults: The National Longitudinal Study of Adolescent to Adult Health (Add Health). *Biomedicines* **2022**, *10*, 2756. https://doi.org/10.3390/biomedicines10112756

Academic Editor: Anna Morgan

Received: 24 September 2022
Accepted: 20 October 2022
Published: 31 October 2022

Publisher's Note: MDPI stays neutral with regard to jurisdictional claims in published maps and institutional affiliations.

Copyright: © 2022 by the authors. Licensee MDPI, Basel, Switzerland. This article is an open access article distributed under the terms and conditions of the Creative Commons Attribution (CC BY) license (https://creativecommons.org/licenses/by/4.0/).

Supplementary Materials: The following supporting information can be downloaded at: https://www.mdpi.com/article/10.3390/biomedicines10112756/s1, **Table S1**: Associations between hearing loss and allele and genotype of rs1800497 and rs1800497; **Figure S1**: Prevalence of hearing loss by allele and genotype of rs1800497; **Figure S2**: Prevalence of hearing loss by allele and genotype of rs12945042.

Table S1. Associations between hearing loss and allele and genotype of. rs1800497 and rs1800497

SNP	Model *		β Estimates (95% CI)	Weight
**rs800497 (*DRD2*)**	**Allele (A vs. G)**			
		Non-Hispanic White	-0.05 (-0.19 - 0.09)	67.8%
		Non-Hispanic Black	-0.06 (-0.34 - 0.22)	16.5%
		Hispanic	-0.23 (-0.58 - 0.13)	10.9%
		Non-Hispanic Asian	-0.15 (-0.68 - 0.38)	4.8%
		Meta-analysis	-0.08 (-0.19 - 0.04)	
		P value	0.1940	
	**Genotype (additive model)** (reference: GG)			
		Non-Hispanic White	-0.06 (-0.20 - 0.08)	67.6%
		Non-Hispanic Black	-0.07 (-0.35 - 0.21)	16.0%
		Hispanic	-0.20 (-0.55 - 0.14)	10.9%
		Non-Hispanic Asian	-0.05 (-0.53 - 0.44)	5.5%
		Meta-analysis	-0.08 (-0.19 - 0.04)	
		P value	0.1899	
**rs12945042 (near *SLC6A4*)**	**Allele (C vs. T)**			
		Non-Hispanic White	0.03 (-0.09 - 0.16)	73.4%
		Non-Hispanic Black	-0.12 (-0.43 - 0.19)	12.2%
		Hispanic	-0.16 (-0.50 - 0.17)	10.4%
		Meta-analysis	-0.03 (-0.14 - 0.08)	
		P value	0.6283	
	**Genotype (additive model)** (reference: TT)			
		Non-Hispanic White	-0.02 (-0.15 - 0.10)	83.8%
		Non-Hispanic Black	0.12 (-0.20 - 0.44)	11.7%
		Hispanic	0.15 (-0.19 - 0.49)	10.2%
		Non-Hispanic Asian	0.47 (-0.05 - 0.99)	4.3%
		Meta-analysis	0.03 (-0.08 - 0.14)	
		P value	0.5501	

- The allelic and genotypic association modelas were evaluated using regression (additive model) adjusted for age, sex, education level, smoking status, and household income by racial/ethnic groups.

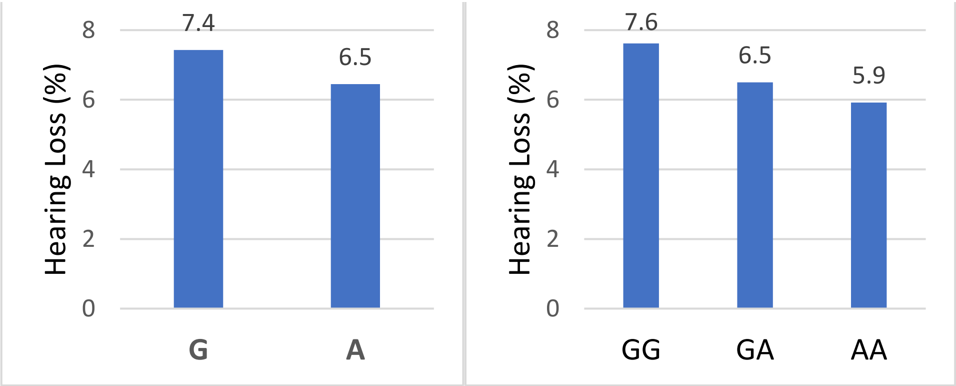

(**a**) (**b**)

Figure S1. Prevalence of hearing loss by allele and genotype of rs1800497 (DDR2): (**a**) allele; (**b**): genotype.

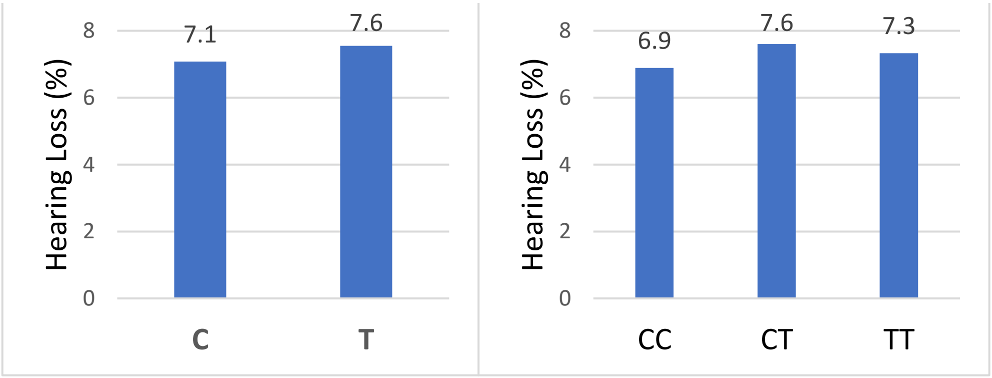

(**a**) (**b**)

Figure S2. Prevalence of hearing loss by allele and genotype of rs12945042: (**a**) allele; (b): genotype.
